# A strategic reflection for the management and implementation of CAR-T therapy in Spain: an expert consensus paper

**DOI:** 10.1007/s12094-021-02757-9

**Published:** 2022-01-08

**Authors:** N. Zozaya, J. Villaseca, F. Abdalla, M. A. Calleja, J. L. Díez-Martín, J. Estévez, R. García-Sanz, J. Martínez-López, J. Sierra, R. Vera, A. Hidalgo-Vega

**Affiliations:** 1 Health Economics Department, Weber Economía y Salud, C/Moreto 17, 5D, 28014 Madrid, Spain; 2 Department of Quantitative Methods in Economics and Management, University of Las Palmas de Gran Canaria, Las Palmas, Spain; 3 Pharmacy Department, Hospital Universitario Virgen Macarena, Seville, Spain; 4 Hematology Department, Hospital GU Gregorio Marañón, Gregorio Marañón Health Research Institute, Madrid, Spain; 5 Department of Medicine, Complutense University of Madrid, Madrid, Spain; 6 The Spanish Society of Health Executives, Sociedad Española de Directivos de La Salud (SEDISA), Madrid, Spain; 7 Hematology Service, CIBERONC, and Salamanca Cancer Research Centre-IBMCC (USAL-CSIC), University Hospital of Salamanca (HUS/IBSAL), Salamanca, Spain; 8 Hematology Department, Hospital Universitario, 12 de Octubre, Complutense University of Madrid, CNIO, CIBERONC, Madrid, Spain; 9 Hematology Department and Sant Pau Biomedical Research Institute (IIB-SP), Hospital de la Santa Creu i Sant Pau, Univarsitat Autonoma of Barcelona, Barcelona, Spain; 10 Medical Oncology Service, Navarra Hospital Complex, Navarra Hospital, Pamplona, Navarra Spain; 11 Weber Foundation, Madrid, Spain; 12 Department of Economic Analysis and Finance, University of Castilla-La Mancha, Toledo, Spain

**Keywords:** Advanced therapies, CAR-T therapy, National health system, Spain

## Abstract

CAR-T cell therapy represents a therapeutic revolution in the prognosis and treatment of patients with certain types of hematological cancer. However, they also pose new challenges in the healthcare, regulatory and financial fields. The aim of the RET-A project was to undertake a strategic reflection on the management of CAR-T therapies within the Spanish National Health System, to agree on recommendations that will help to better deal with the new context introduced by these cell therapies in the present and in the future. This think tank involved 40 key agents and opinion leaders. The experts identified three great challenges for implementing advanced therapies in Spain: therapeutic individualisation, with a multidisciplinary approach; acceleration of access times, by minimizing bureaucracy; and increase in the number of centers qualified to manage the CAR-T therapies in the NHS. The experts agreed on the ideal criteria for designating those qualified centers. They also agreed on a comprehensive CAR-T care pathway with the timings and roles which would ideally be involved in each part of the process.

## Introduction

In the past decade, advances in cellular and molecular research have led to the development of CAR T-cell therapy (chimeric antigen receptor T-cell therapy), also known as CAR-T. It is a new generation of personalized immunotherapy which represents a therapeutic revolution in the prognosis and life expectancy of patients with certain types of cancer. However, this therapy also poses new challenges in the healthcare, regulatory and financial fields, which it is necessary to know to anticipate them and optimize their approach in the healthcare system.

The aim of this study is to detail the RET-A project, ‘A Strategic Reflection for the management and implementation of the new Advanced Therapies in Spain’, which gathered experts together to reflect about the main challenges, opportunities and strengths of the Spanish NHS regarding CAR-T and to agree on recommendations to better deal with this new scenario in the present and the future.

## Methodology

As a think tank on CAR-T therapies, the RET-A project brought together the multidisciplinary work of 40 key agents of the Spanish NHS. RET-A was set in motion by an advisory committee (the CORE group), which provided the overview, design and scope of the project, as well the subjects to be dealt with by each working group, and the composition of those groups.

Four thematic multidisciplinary working groups (WG-1, WG-2, WG-3 and WG-4) (Table 1) were constituted. Face-to-face meetings were held among the different WG to share experiences and opinions about concrete important aspects of the CAR-T therapies, to obtain reflections and recommendations for improvement in each area.

**Table 1:** Composition of the working groups participating in the RET-A project

Working groups			
CORE group and WG-1	WG-2	WG-3	WG-4
Joaquín Estévez Lucas. President of the Spanish Society of Health Directors (SEDISA) Joaquín Martínez López. Head of the Hematology Department, ‘12 October’ University Hospital, Madrid José Luis Díez Martín. Head of the Hematology Department, ‘Gregorio Marañón’ University General Hospital, Madrid Jordi Sierra Gil. Head of the Hematology Department, ‘Santa Creu i Sant Pau’ Hospital, Barcelona Miguel A. Calleja Hernández. President of the Spanish Society of Hospital Pharmacy (SEFH), and Head of the Pharmacy Department of ‘Virgen Macarena’ University Hospital, Sevilla Ramón García Sanz. President-elect of the Spanish Society of Hematology and Hemotherapy (SEHH); and hematologist at the University Hospital, Salamanca Ruth Vera García. President of the Spanish Society of Medical Oncology (SEOM); and Head of the Oncology Department, Hospital Complex, Navarra	Ana Lozano Blázquez. Director of the Unit of Clinical Pharmacy Management, Central University Hospital, Asturias Anna Sureda Balari. Head of the Clinical Hematology Department, Catalan Institute of Oncology Ana Rodríguez Cala. Director of Strategy and Corporate Social Responsibility, Catalan Institute of Oncology Antonio Pérez-Martínez. Head of the Pediatric Hemato-Oncology Department, ‘La Paz’ University Hospital, Madrid Begoña Barragán García. President of the Spanish Group of Patients with Cancer (GEPAC), and the Spanish Association of Patients with Lymphoma, Myeloma and Leukemia (AEAL) Emilio Vargas Castrillón. Head of the Clinical Pharmacology Department, ‘San Carlos’ University Clinic Hospital, Madrid Fermín Schez-Guijo Martín. Head of Cell Therapy of the Hematology Department, University Hospital, Salamanca Jaime Masjuan Vallejo. Head of the Neurology Department, ‘Ramón y Cajal’ University Hospital, Madrid Luis de la Cruz Merino. Vice-president of GETICA, and Head of the Medical Oncology Department, ‘Virgen de la Macarena’ University Hospital, Sevilla Patricia Muñoz García. Head of Section in the Infectious Diseases and Microbiology Department, ‘Gregorio Marañón’ University General Hospital, Madrid Pedro Castro Rebollo. Consultant in the Intensive Surveillance Area, Clinic Hospital, Barcelona Sonia García de San José. Deputy Managing Director, ‘Gregorio Marañón’ University General Hospital, Madrid	Antoni Gilabert Perramón. Director of the Pharmacy and Medicine Area of the Health and Social Consortium of Catalonia Francisco Javier López Jiménez. Head of the Hematology-Hemotherapy Department. ‘Ramón y Cajal’ University Hospital, Madrid Francisco Ayala de la Peña. Head of Section of Medical Oncology, ‘Morales Meseguer’ University General Hospital, Murcia José Francisco Soto Banel. Manager Director, ‘San Carlos’ University Clinic Hospital, Madrid José Luis Poveda Andrés. Head of the Pharmacy Department, ‘Universitari i Politècnic la Fe’ Hospital, Valencia Juan Oliva Moreno. Associate Professor, Economic Analysis Department, Castilla-La Mancha University María Antonia Mangues Bafalluy. Director of the Pharmacy Department, ‘Santa Creu i Sant Pau’ Hospital, Barcelona M. Ángel Casado Gómez. General Director of PORIB Pedro Gómez Pajuelo. General Secretary, Spanish National Transplant Organization Sandra Flores Moreno. Hospital Pharmacist, ‘Virgen del Rocío’ Hospital, Sevilla	Bernat Soria Escoms. Researcher at the Bioengineering Institute and Professor at the Faculty of Medicine of ‘Miguel Hernández’ University Carlos Solano Vercet. Head of the Hematology and Hemotherapy Department, University Clinic Hospital, Valencia César Pascual Fernández. Head of the Healthcare Quality Department, User Service and Evaluation of Information, Cantabrian Health Service Cristina Avendaño Solá. Physician specialist in Clinical Pharmacology, ‘Puerta de Hierro’ Hospital, Majadahonda, Madrid Enrique Castellón Leal. President of ‘Cross Road Biotech Inversiones Biotecnológicas’ Felipe Prósper Cardoso. Director of the Cell Therapy Area and Co-Director of the Clinical Hematology Department, University of Navarra Francesc Bosch Albareda. Principal Researcher of the Experimental Hematology Group of the VHIO and Head of the Department of Hematology, ‘Vall d’Hebron’ University Hospital, Barcelona Josep Torrent-Farnell. Deputy Physician and Co-ordinator of Minority Diseases and Orphan Drugs, ‘Santa Creu i Sant Pau’ Hospital, Barcelona Julio Sánchez Fierro. Lawyer and Doctor of Health Sciences María Victoria Mateos Manteca. Hematologist, University Clinic Hospital, Salamanca Natacha Bolaños Fernández. Regional Manager for Lymphoma Coalition in Europe *Guests from the CORE group: Jordi Sierra and Miguel Ángel Calleja

*WG* working group

*WG4 was composed of 11 experts, and 2 experts of the CORE Group also attended the meeting as guests

To prepare the meetings, a detailed review of information about the different subjects connected with the CAR-T therapy was performed. It included, among other issues, a description of CAR-T therapies, the Spanish Ministry of Health’s plan for advanced therapies, the evaluation and financing of CAR-T therapies, and follow-up mechanisms. The project was led by Weber (a center for Research and Consulting in Health Economics), who dealt with the search for information, the review of the literature, as well as the organization and moderation of each group of experts.

## Relevant aspects in CAR-T therapies: literature review

### A description of CAR-T therapies

In 2018, the European Medicines Agency (EMA) and the U.S. Food and Drug Administration (FDA) authorized the marketing of the first two industrially produced drugs with CAR T-cells (tisagenlecleucel and axicabtagene ciloleucel) [1–4]. They provided promising clinical results to satisfy unmet therapeutic needs among patients with B-cell acute lymphoblastic leukemia (B-cell ALL) and diffuse large B-cell lymphoma (DLBCL), with relapse or who had not responded to the available alternative treatments after two or more lines of therapy [5–7].

Before these therapies were approved, only palliative or experimental treatments were applied. In patients with refractory B-cell ALL, the only potentially curative option was the allogeneic hematopoietic cell transplant (allo-TPH), whose overall survival rate at 5 years was 20–45% [8, 9]. In cases of relapse after an allo-TPH, there was no standard treatment, and the palliative treatments had an average survival period of less than 8 months [10, 11].

However, there is uncertainty about the real, long-term effectiveness of these advanced therapies that are tailor-made for each patient, require a multidisciplinary approach [12–14], as well as certain conditions for the caring capacity, and availability of human and technical resources in the medical centers to ensure that their administration is reliable and safe [15]. Moreover, these therapies are costly, so different financing schemes were set to promote their access [16–20]. For all these reasons, the management of CAR-T therapy is different from that of drugs aimed at therapeutic targets and requires a more complex care procedure [12].

### The Spanish Ministry of Health’s plan for advanced therapies

In 2018, the Spanish Ministry of Health approved, within the precision medicine strategy, the ‘Plan for Approaching Advanced Therapies in the National Health System: CAR Medicines’, aimed to organize in a planned, equitable, safe and efficient way the use of these therapies [12]. Different stakeholders participated in its design and development.

The plan proposed an organizational model comprising a network of qualified centers for the use and administration of CAR medicines—currently, 14-, based on certain criteria [21, 22]. These centers, located in six Autonomous Communities, have to attend all patients on equal terms regardless of their place of residence, provide care in a multidisciplinary team, guarantee continuity in care between stages of the patient's life and between levels of care, evaluate the results and train other professionals. The plan also considered the creation of a committee at the national level to quickly assess the requests made by NHS specialists, whose favorable report will be mandatory for the use of the medicine.

The strategy of the Ministry of Health in advanced therapies had a dynamic character to evaluate and monitor the plan, identifying points to improve. And it will use horizon scanning to identify the CAR-T therapies that are being incorporated in the medium / long term and the needs to be satisfied [23]. To date, three follow-up reports have been published [24–26]. Some Autonomous Communities have also tried to standardize and streamline the process of CAR-T therapy [27–30].

### Assessment and price reimbursement of CAR-T therapies

The current assessment of the overall value associated with most innovative healthcare technologies, including advanced therapies, has to deal with the difficulty of producing, at the time of commercialization, plausible evidence to reach the standards required to obtain national reimbursement [31]. Besides the possibility of curing the patients, the benefits associated with the CAR-T therapies also reach the patients’ carers, their relatives and society in general [32]. Much of their value can derive from savings due to treatments and procedures avoided, and from improvements in patients’ quality of life and productivity at work, throughout their lives.

The two first industrial CAR-T therapies have been rapidly and positively evaluated by several agencies for Health Technology Assessment (HTA), although the decisions were not always unanimous [33]. The UK recommended the two CAR-T therapies for all their indications (B-cell ALL, DLBCL), through the ‘Cancer Drugs Fund’ [34–36]. Scotland recommended the use of tisagenlecleucel for B-cell ALL, but not for DLBCL, whereas it did recommend axicabtagene ciloleucel for DLBCL [37–39]. France assessed the degree of innovation of tisagenlecleucel for B-cell ALL as grade III. For DLBCL, France awarded a lower grade (IV) to tisagenlecleucel than to axicabtagene ciloleucel (III) [33].

The approval of treatments that are potentially curative, such as the CAR-T therapies, gives rise to more challenges for healthcare systems because the payment of the associated costs has to be made at the beginning of the therapy (which is administered only once), whereas the greatest benefits are obtained in the long term. This situation is, therefore, very different from that of other oncological therapies which are administered over a prolonged period. There is, therefore, an obvious need for new approaches which offer payers a system that reduces the complications associated with the financing of these therapies, at the same time facilitating rapid access to patients who need them, and also ensuring that the system is sustainable.

CAR-T therapies are costly, due not only to the costs of research and development but also to the complexity of the processes of manufacturing and administration. Therefore, the national authorities of several countries, including Spain, have linked CAR-T therapies to systems of payment by results. Besides, some countries have provided funds specifically for these or other innovative therapies, such as the ‘Cancer Drugs Fund’ of the United Kingdom [40] or the fund for innovative oncological medicines in Italy [41]. In Spain, the drug’s costs are borne in full by the patient’s local hospital, and the costs of care are advanced by the Autonomous Community in which the treatment will take place, and will be subsequently refunded by the Health Cohesion Fund [42, 43].

### Follow-up mechanisms

The management of CAR-T therapies requires long-term follow-up of results in terms of effectiveness and adverse effects, involving the compiling of data from Real World Evidence (RWE). These data also allow the compilation of information about the quality of life associated with the health of the treated patients, and the healthcare resources used to implement the therapy. EMA lays down, as a condition of the authorization to market the CAR-T therapies, the need for keeping a detailed record of the clinical data of the candidate patients and of those who have received the drug [44].

There are several European initiatives for harmonizing and optimizing the RWE’s records on advanced therapies, including recommendations for improvements such as those proposed by the Alliance for Regenerative Medicine [31], EMA [45] and European Network for Health Technology Assessment (EUnetHTA) [46].

In Spain, the Ministry’s Plan envisaged the use of the new VALTERMED (*Valor Terapéutico del Medicamento*: Therapeutic Value of Medicines) information system to determine the therapeutic value, in actual clinical practice, of some drugs, including those used in CAR-T therapies, that have a high impact on healthcare and finances within the NHS.

## Reflections and recommendations from the RET-A project

### Working group 1 (WG-1): strategic starting map

WG1—composed of seven experts from the clinical and managerial fields and from four scientific societies, who also composed the CORE Group—reflected on the current situation of the CAR-T therapies in Spain, and proposed, based on the Ministry’s Plan on CAR-T, strategic actions for improvement in the short and long terms.

#### Reflections

The group reviewed the strategic starting point, including the Plan’s strengths and possibilities for improvement. The RET-A experts agreed that the Ministry’s plan was very necessary, well designed, and had well-justified objectives. They praised that it tried to anticipate real clinical practice, involving many of the agents of the system. In addition, they enhanced its dynamic character, which will make it easier for it to be updated over time.

Moreover, the WG reflected upon the ideal criteria for designating centers qualified to manage the CAR-T therapies in the NHS, versus the official ones contained in the Ministry’s plan (Table 2). In this sense, for the administration of commercial CAR-T, some specific aspects could be ignored, such as the capacity for cell production in situ or the availability of a specific CAR-T committee. Similarly, they mentioned that the activity in complex allogeneic transplants could be assessed, although the need for it to be a requirement was not seen as necessary. In addition, some of the experts noted that the follow items could be assessed as additional but mandatory merits; having JACIE accreditation, promoting territorial equity (qualification in a region without a designated center), and evaluating the capacity of the hospital pharmacy of the center.

**Table 2:** Possible lines of advance in the ‘Plan for Approaching Advanced Therapies in the National Health System: CAR Medicines’

Current criteria	Alternative proposal of criteria
Total activity of allogeneic hematopoietic stem cell transplants in the past 3 years Total activity of complex allogeneic hematopoietic stem cell transplants in the past 3 years To have the JACIE-CAT-ONT accreditation To have accreditation as CSUR for pediatric units Known/demonstrable complex cell processing activity To have a Cell Therapy Unit/Area or multi-disciplinary units created for this purpose Previous clinical or preclinical experience with CAR-T medicines Capacity to manufacture own CAR-T medicines: authorization from the AEMPS for manufacture “in house” Total apheresis activity of stem cells Provision of a clinical-pathological and multidisciplinary committee to review candidate cases for CAR-T therapies, as well as experts in the pathologies for which it is indicated	Total activity of allogeneic transplants of stem cells, including complex ones, in the past 3 years Previous clinical and preclinical experience with CAR-T medicines, with the same weight for both types Total apheresis activity of stem cells To have a cell therapy unit or multi-disciplinary units (they do not need to be created for this purpose, nor do they need to be specific for CAR-T). The ideal would be to spend the same day of the unit meeting to do 2 parts: first for the diagnosis of new patients and then for the treated cases As an additional merit: To have JACIE-CAT-ONT accreditation (laboratory and clinic) or to have requested it and be still in the approval process Territorial/population equity Hospital pharmacy capacity: 24-h accessibility, experience with advanced therapies

AEMPS (Agencia Española de Medicamentos y Productos Sanitarios): Spanish Agency for Medicines and Health Products. CSUR: Reference Centers, Departments and Units

Another aim of this group was to outline the possible locations of additional qualified centers to manage the CAR-T therapies in the NHS, in the short and long term. The experts considered the following premises: (1) In general, one center will be necessary for every 2 million inhabitants; (2) For the short-term target population, the two indications already approved for industrial CAR-T therapy were considered: about 200 patients per year with B-cell ALL and about 800 per year with DLBCL. (3) The target population at 5 years was estimated at about 1500 patients per year, based on oncohematological indications for CAR-T therapy that have already been approved, as well as new CAR-T for other oncohematological diseases and solid tumors. When preparing the maps, consideration was given to the population of each Autonomous Community, the distance from other regions, the centers where allogeneic and/or autologous transplants were performed, the centers’ capacity for caring, and the current and future target populations.

#### Recommendations

**Fig. 1:**
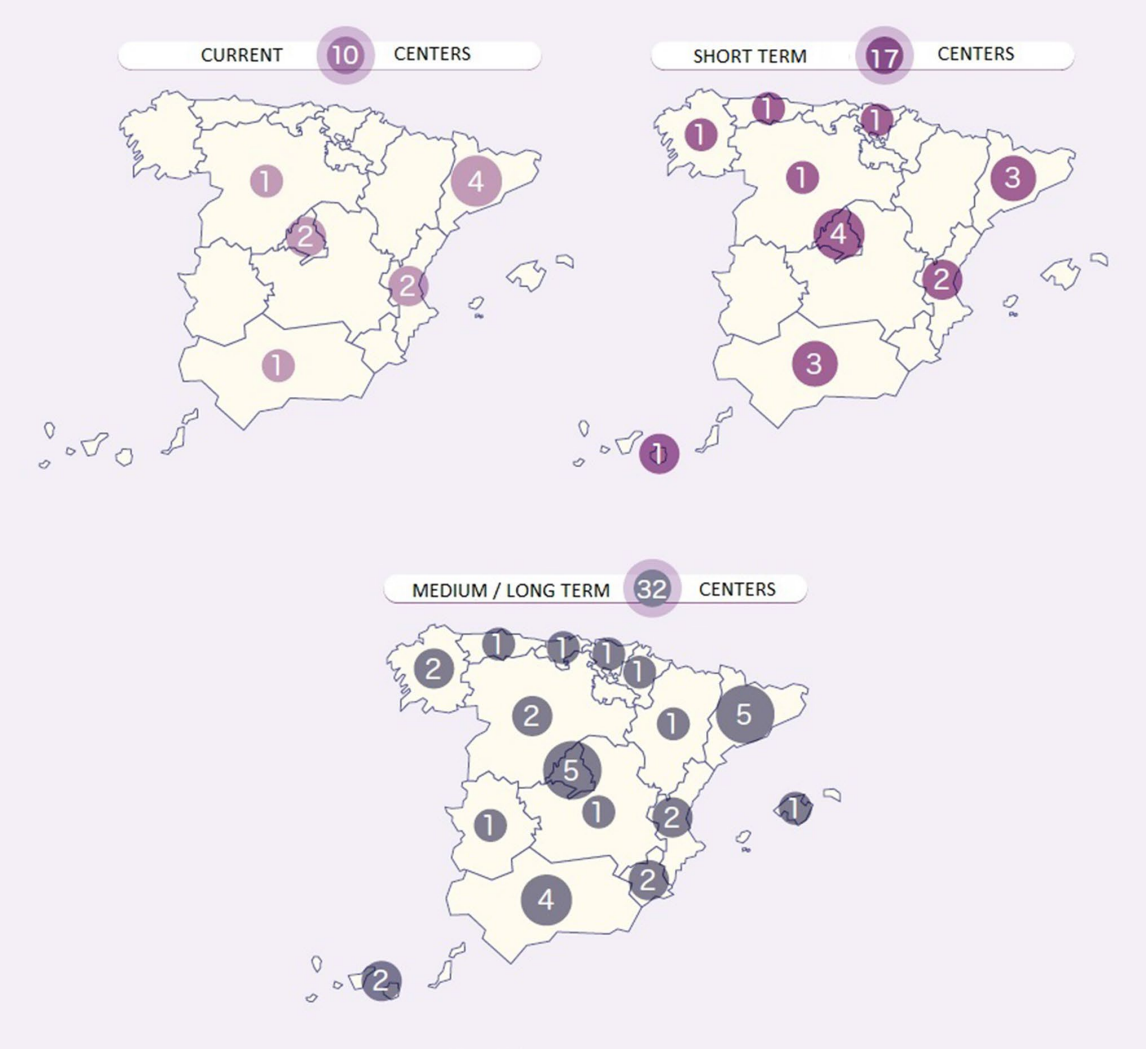
Maps of current and ideal locations of qualified CAR-T centers in the short term (17 centers) and medium/long term (32 centers)

The experts proposed the following recommendations:

- It would be desirable to extend the number of CAR-T qualified centers to move from the actual 14 centers to 17 in the short term and 32 centers in the medium term (Fig. 1). Speeding up of the procedures needed to approve CAR-T treatments, simplifying the stages and committees involved at the ministerial, regional and hospital levels.
- To streamline the process, the CAR-T evaluation committees should be unified.
- The management of the CAR-T therapies is going to foster a cultural change, as it involves multidisciplinary work. Common standards are needed, to avoid excessive prominence of individuals and to encourage teamwork.
- The healthcare managers have to be involved in the process and need to have the ability to give appropriate financial and legal advice. They need to be facilitators, coordinators and evaluators, with a fundamental role in the re-organization of hospital procedures and in impelling the training of the clinicians. The healthcare managers also need to receive training (and updates).
- It would be advisable that the center of origin of the patient establishes an order of priority when choosing the centers that will administer the CAR-T therapies.
- Information should flow as smoothly as possible between the qualified hospitals and the others, as well as between the Autonomous Communities, the Ministerial committees, and the reference centers.
- It will be necessary to insist on strategic co-ordination nationally, and hence among the Autonomous Communities, to make interoperability viable and to ensure that data are shared instead of being kept in niches in each hospital.
- Degrees of training and commitment differ among hospitals. There needs to be adequate training of the staff who manage the CAR-T therapies (the ‘CAR-T-ologists’) and of everyone involved in the treatment and the follow-up.
- The different CAR-T therapies are not equally effective, so we must not equate them to one another or take their contributions for granted. Instead, we must evaluate the results actually achieved.
- As soon as the drug is approved, the industry must study purchasing proposals which make it viable to introduce it into the healthcare system.
- In Spain, there should be a SIFCO (Information System of the Health Cohesion Fund) specifically for CAR-T, or a similar fund, providing financing commensurate with the cost of the therapy.
- The triple reporting of data should be simplified by means of a unified tool which could be used for the different databases.
- The authorities should make an effort to transfer the centers of production of CAR-T to Spain, based on the example of France and the UK.

### Working group 2 (WG-2): the comprehensive care process

WG-2—composed of 12 experts from the clinical, caring and managerial fields and a patients’ representative—reflected on the need to give comprehensive consideration to the care process in the CAR-T therapies within the NHS. The group held discussions in relation to ‘Roles and resources’ and ‘Training, Communication, Co-ordination’ to propose a comprehensive care pathway with ideal timings.

#### Reflections: ‘Roles and resources’

In the reflection on ‘Roles and resources’, this group of experts considered that there was a need for a multidisciplinary team including specialists in Hematology, Pediatric Oncohematology (in cases of children), Oncology (in oncological patients), Intensive Medicine, Neurology, Clinical Pharmacology, Immunology, Infectology, Radiology, Hospital Pharmacy, Clinical Research, Pathology, Nursing and Medical Management, as well as other social or healthcare experts and the patient’s relatives.

#### Recommendations: ‘Roles and resources’

They also proposed the following recommendations:

- Determine who should lead the multidisciplinary group.
- Create the figure of the ‘case manager’ in CAR-T; this role could be assumed by nursing or other social/healthcare personnel. The patients could rely on this person as a source of information and orientation about the care process.
- Create the figure of the ‘data manager’. A lot of information is generated about this therapy (through the entry of data into company platforms, collection of clinical data, etc.), and having a specialized data manager available would allow healthcare workers to lighten their workloads.
- Define and standardize the way to perform necropsies on patients who have died while undergoing treatment with a CAR-T therapy.
- Adequately plan the structural resources so that the process is well integrated within the hospital.
- Create cellular production units. This would ensure a return on the investment.
- Standardize the information systems, using a single database to avoid the duplication of data.
- Create an accreditation system that is common to all the centers.

#### Recommendations: ‘Training, Communication and Co-ordination’

The experts focused their discussion on recommendations regarding training, communication and co-ordination. It would be desirable to:

- Improve the training of the multidisciplinary team that manages these therapies, including nurses and managers.
- Maintain a regular training of the leaders who teach other specialists, with international references, by means of congresses, research, visits to foreign centers, etc.
- Candidate patients for CAR-T therapy, and their relatives, should also receive training.
- Improve the training of the media professionals, so that scientific journalism is of high quality.
- Improve intra-hospital co-ordination. To achieve that, it would be necessary to identify all the agents involved (the multidisciplinary team), to define their responsibilities, and to provide them with information about the procedures to be followed.
- Improve co-ordination among hospitals.
- Streamline bureaucratic procedures and avoid errors.
- Have standards of quality and patients’ safety in place, with specific indicators. For this, the figure of the ‘data manager’ is required. The data collected should also be used for research, for improving the care process, and for accounting.
- Provide the patients with adequate information.
- Set up a system (perhaps telephonic) for knowing, in real time, what options can be given to the patient: and, if the patient is a candidate for CAR-T therapy, which centers are accredited for caring for that patient and what clinical trials are available in Spain at that time.
- Improve transparency and accountability to society.
- Improve co-ordination between the NHS and the pharmaceutical industry.

**Fig. 2:**
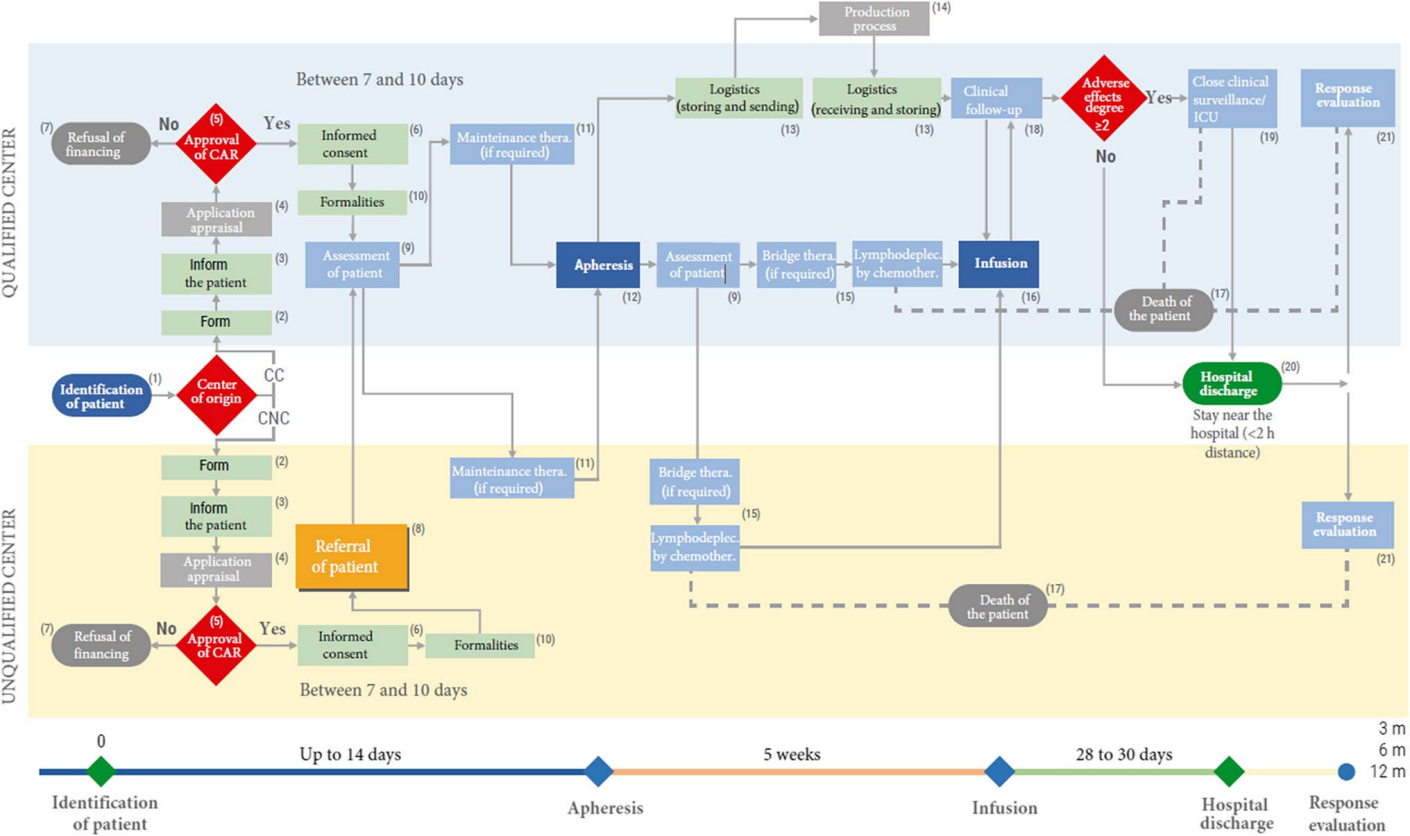
Comprehensive care circuit for CAR-T therapy proposed by Working Group 2 of RET-A (more detailed information in Table 3)

**Fig. 3:**
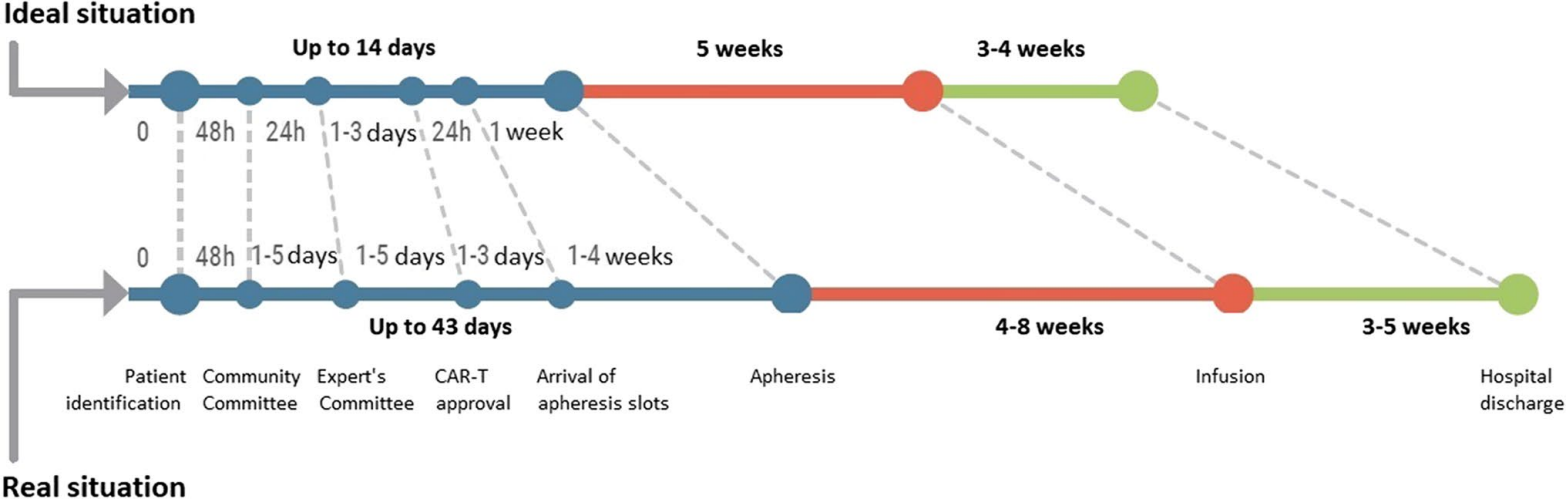
Real versus ideal times at each stage of the CAR-T in Spanish care management circuit

Finally, as an output to the discussion held*,* a detailed comprehensive care pathway was recommended (Fig. 2 and Table 3). WG-2 also reviewed the current situation and the timings that would be desirable in the different stages of the care pathway of the CAR-T therapies (Fig. 3). Although there was considerable heterogeneity between centers, the patient must wait up to 6.1 weeks from the moment when he/she is identified as a candidate for these therapies until apheresis is finally performed (or 10 weeks if his/her center of origin is not qualified). The times required by the different phases of the CAR-T care pathway were still far from the optimum that could be achieved. It was estimated that there was a margin of improvement of 42% in qualified centers and of 52% in unqualified ones, since the total times of the process (between the identification of the patient and their discharge from hospital) could be reduced by 8.1 and 11.9 weeks, respectively.

**Table 3:** Agents of oncohematological indications involved in each activity within the CAR-T comprehensive care circuit (proposed by working group 2 of the RET-A project, represented in Fig. 2 of the article)

No.	Activity	Agents involved
(1)	Patient identification	Hematologist/oncologist/pediatric oncohematologist
(2)	Form	Hematologist/oncologist/pediatric oncohematologist
(3)	To inform the patient	Hematologist /pediatric oncohematologist; patient; relatives; Nursing
(4)	Evaluation of the request	Group of experts in CAR-T from Hospital/Autonomous Communities/Committee of experts of Ministry
(5)	CAR-T approval	Committee of experts of Ministry
(6)	Informed consent	Hematologist /pediatric oncohematologist; patient; relatives; Nursing
(7)	Rejection of financing	Committee of experts of Ministry
(8)	Referral of the patient	Non-designated center: hematologist/pediatric oncohematologist; medical management. Designated Center: CAR-T Hospital
(9)	Assessment of the patient	Hospital group of experts in CAR-T: hematologist, oncologist, intensivist, pediatric oncohematologist, infectologist, neurologist, radiologist, immunologist, pharmacologist, clinician, clinical investigator, and other specialists as required; medical management; hospital pharmacy; Nursing. social worker, psychologist
(10)	Formalities	Patient; relatives; social worker, psychologist
(11)	Maintenance treatment	Hematologist/pediatric oncohematologist/oncologist; Nursing; clinical pharmacologist; hospital pharmacy
(12)	Apheresis	Blood Bank/Cell Therapy Unit; Nursing; hospital pharmacy; case manager; data manager
(13)	Logistics	Blood Bank/Cell Therapy Unit; Nursing; hospital pharmacy; case manager; data manager
(14)	Production process	Laboratory/Pharmaceutical Industry; Cell production units
(15)	Lymphodepletion (if required)	Hematologist/pediatric oncohematologist/oncologist; Nursing; clinical pharmacologist; hospital pharmacy
(16)	Infusion	Blood Bank/Cell Therapy Unit; Nursing; hospital pharmacy; case manager; data manager
(17)	Death of the patient	Pathology; clinical researcher; relatives
(18)	Clinical follow-up	CAR-T Hospital group of experts: hematologist, oncologist, intensivist, pediatric oncohematologist, infectologist, neurologist, radiologist, immunologist, pharmacologist, clinician, clinical investigator, and other specialists as required; hospital pharmacy; Nursing; medical management; case manager; data manager
(19)	Surveillance/Clinical follow-up	CAR-T Hospital Expert Group: hematologist; oncologist; intensivist, pediatric oncohematologist, infectologist, neurologist, radiologist, immunologist, clinical pharmacologist, clinical investigator and other specialists as required; hospital pharmacy; Nursing; medical management
(20)	Hospital discharge	Hematologist/pediatric oncohematologist; patient; relatives
(21)	Response evaluation	Hematologist/pediatric oncohematologist; case manager; data manager. The times for evaluating the response to therapy depend on the indication. In lymphoma: quarterly follow-up in the first year, semi-annually in the second, and annually thereafter. In ALL: bi-weekly follow-up until the third month, monthly until the sixth month, and bi-monthly until the first year

### Working group 3 (WG-3): innovative models for appraisal and financing

WG-3—composed of ten experts in the financial, clinical, academic and managerial fields—reflected on the innovative models needed for the evaluation and financing of CAR-T therapies. The experts’ objective was to make recommendations for improvement in the processes of economic evaluation, financing and follow-up of the CAR-T therapies in Spain, to better prepare the system for the personalized therapies to come.

#### Reflections

According to the experts, when taking decisions, one should not disregard economic evaluations. If the actual results are evaluated using simple variables, the benefit for patients and the amount that has to be paid for it can be ascertained. Raising civil society’s awareness, by means of a scientifically validated methodology, should allow all of us to limit the effort that needs to be made in relation to these therapies and the results that we expect from them.

They also pointed out that the financing, together with the results, is the first point to be considered in the approach to these therapies, and is irrevocably linked to the quality of the follow-up of the process. Failing in operations as simple as the collection of data could jeopardize all advances in innovative financing schemes. Therefore, an intelligent and rigorous approach to the monitoring of the health results is the cornerstone of the financing and follow-up of these therapies.

#### Recommendations

The group proposed the following actions:

- Requirement to carry out economic evaluations of advanced therapies, based on a clear methodology, to inform financing decisions and drug pricing.
- Economic evaluations using the public payer perspective and, secondly, the social perspective.
- Payments by results must be made in accordance with clear and transparent criteria.
- Create, in the medium term, several pre-designed models of agreements for payment by results and by indication.
- It is necessary to inform society about and involve it in all the aspects which the new therapies represent for healthcare.
- Possibility of identifying a potential specific finalist fund for emerging therapies.
- Harmonize the health and economic impact variables of the national registry with the EBMT.
- Enhance the quality and credibility of the data collected in the clinical history, with automatic information capture systems.
- Improve the EBMT registry, starting with disease working groups.
- Systematic representation of patients to design what data, including PRO, it would be advisable to collect. Accessibility to the registration systems by patients should also be improved.
- Creation of the figure of the data manager.
- The professional should be made more involved in the achievements, receiving feedback of comparative data.

### Working group 4 (WG-4): reflection on the future projection

WG-4—composed of 11 experts from the clinical, managerial, political and technological fields, complemented, as guests, by two experts from the CORE group—reflected on the future, in an attempt to predict the way in which the CAR-T therapies will evolve, and their needs in the medium and long terms. The group evaluated the scalability of the current structure, processes, models and systems, based on the therapies which are still to come in hematology, oncology and other specialties.

The group focused on the following aspects: the patient’s perspective, the care process, the regulation and financing, the information systems, and the research. The group also performed a SWOT analysis (strengths, weaknesses, opportunities, and threats) of the way in which the NHS will accommodate these therapies in the future.

According to the experts, new therapies, and in particular CAR-T, are a factor that contributes to the transformation of the NHS, which needs a complete review. CAR-T must be an opportunity for the NHS to really re-orientate itself towards its fundamental goals, which are equity and efficiency. In this sense, CAR-T therapy is an opportunity for patients, health professionals and society to improve healthcare processes, increasing their effectiveness and efficiency, and promoting a culture of evaluation of results. This therapy is promising but requires further collaboration, consolidation and adaptation to innovation. We are facing a new paradigm; a new opportunity to re-launch clinical research and caring.

The future of CAR-T therapies is promising and exciting, but we need to be agile when incorporating innovation and prioritizing reassessment. In the future, to serve many specialties, CAR-T services have to be a central service as the transfusion or blood analysis services are now. Changes are needed in the development model, prioritizing active public–private collaboration in research, caring and resources. We also have to invest in relevant tools, such as artificial intelligence.

## Discussion and conclusion

RET-A is a pioneering project in Spain, conceived as a forum for strategic debate on CAR-T therapies. It is an opportunity to optimize the management of these advanced therapies and to learn from the experts with the most experience in this field. The National Plan is a good and needed first step to adapt the management of the therapies to real needs, speeding up times and focusing knowledge in a reduced number of qualified centers of CAR-T in Spain.

However, following the approval of the first industrial CAR-T’s in oncohematology, further approvals are expected in the future in other indications, as well as in solid tumors and even other non-oncological diseases [47, 48]. Therefore, it is important to prepare the system to face the future. This need was exacerbated during 2020 due to the COVID-19 pandemic that affected the continuity of essential routine healthcare services and procedures [49].

The current structure of qualified centers for the management of CAR-T within the NHS has made it possible to concentrate efforts and experiences in a reduced number of centers of excellence. However, for reasons of territorial equality, it will be necessary to rethink the dimensioning of the current healthcare structure, and possibly extend the management of CAR-T to a greater number of centers, especially in those regions geographically far from qualified centers.

Combining equity and territorial equality with agility in patient access is also an important challenge, for which it will be key to minimize bureaucracy, automate processes as much as possible, and enhance communication. In this changing reality, we must learn from the mistakes and successes of others, shortening times and optimizing the selection and referral phases of patients. In addition, the patient must be more clearly involved in all stages of the process, from their adequate informed consent to the collection of data on their reported health outcomes.

The management of CAR-T must necessarily be multidisciplinary, with a process that, placing the patient at the center of the system, allows interaction between the different medical specialties, as well as the hospital pharmacy, nursing and medical management, among others. For this, the processes must be optimized, with joint training efforts—which must be individualized and continuous—and communication, seeking the involvement of all the parties involved.

CAR-Ts are very promising treatments for patients who lack therapeutic alternatives. However, it is convenient to differentiate the therapies based on their efficacy, safety, uncertainty, and cost-effectiveness. In general, it is necessary to associate its commercialization with financing schemes based on the results obtained in real life, with a longer-term vision of traditional annual budgets. The subsequent evaluation of the results obtained from the information systems will be essential for the reimbursement of the therapies and to be able to continue making decisions based on evidence and value. The challenge in this regard lies in making the professional a part of the achievements, without adding a substantial burden. For this, it will be key to harmonize and simplify the information systems, seeking for the interoperability of the different databases.

Maximum use, both of current therapies and those yet to come, must come from public–private collaboration. All parties must accept this binomial scenario without entering into the competition and moving towards the central axis of any health system, which is the improvement of the health of all patients.

In conclusion, this think-tank provided concrete recommendations on the management of CAR-T therapies, to optimize their use and make the best possible use of the available public resources. Further research will be needed to assess how changes are implemented over time and how they impact the system’s equity and efficiency.
